# Crystal structure of (μ-*trans*-1,2-bis­{2-[(2-oxido­phen­yl)methyl­idene]hydrazin-1-yl­idene}ethane-1,2-diolato-κ^3^ *O*,*O*′,*N*)bis­[di-*tert*-butyl­tin(IV)]

**DOI:** 10.1107/S2056989018007077

**Published:** 2018-05-15

**Authors:** Cheikh Ndoye, Waly Diallo, Ousmane Diouf, Aliou Hamady Barry, Mohamed Gaye, Romain Gautier

**Affiliations:** a Département de Chimie, Faculté des Sciences et Techniques, Université Cheikh Anta Diop, Dakar, Senegal; b Département de Chimie, Faculté des Sciences, Université de Nouakchott, Nouakchott, Mauritania; c IMN Institut ds Matériaux Jean Rouxel, 2 rue de la Houssiniere, 44322 Nantes, France

**Keywords:** crystal structure, tin, Schiff base

## Abstract

The binuclear complex, [Sn_2_(C_4_H_9_)_4_(C_16_H_10_N_4_O_4_)], contains two Sn^4+^ ions, connected by doubly N-deprotonated oxalylbis[(2-oxido­benzyl­idene)hydrazide] ligands, and each Sn^4+^ ion is linked to two *tert*-butyl groups. The coordination sphere of each Sn atom is best described as a distorted trigonal bipyramid. Each stannic ion in the complex is in a C_2_O_2_N environment. The two homologous parts of the doubly deprotonated ligand are located in *trans* positions with respect to the C—C bond of the oxalamide group. The oxalamide group exhibits an asymmetric coordination geometry, as seen by the slight difference between the C—O and C—N bond lengths. The three-dimensional network is a multilayer of complex mol­ecules with no strong supramolecular inter­actions.

## Abstract 2

In a binuclear complex containing two Sn^4+^ ions, connected by the doubly N-deprotonated oxalylbis[(2-oxido­benzyl­idene)hydrazide] ligands, and with each Sn^4+^ is linked to two *tert*-butyl groups, the coordination sphere of each Sn atom is best described as a trigonal bipyramid.

## Chemical context   

Stannic Schiff base complexes formed using a salicyl­aldehyde derivative as a keto precursor have been widely studied in recent decades (Reisi *et al.*, 2010 ▸; Kumar & Nath, 2018 ▸; Tan *et al.*, 2017 ▸; Paul *et al.*, 2014 ▸; Pérez-Pérez *et al.*, 2016 ▸). These Schiff bases may have both hard-atom donors, such as nitro­gen or oxygen (Stadler *et al.*, 2009 ▸; Rehman *et al.*, 2008 ▸; Yin *et al.*, 2008 ▸), and/or soft-atom donors, such as sulfur (Hong *et al.*, 2010 ▸), which allow them to bind to different types of metal ions, yielding complexes with inter­esting properties. Due to the ability of the Sn^4+^ ion to form very stable complexes with Schiff bases or carbanions, many studies have been carried out with regard to their potential applications in medicine (Beltrán *et al.*, 2007 ▸), catalysis (Orita *et al.*, 1999 ▸) and biotechnology (Pellerito & Nagy, 2002 ▸). Schiff bases with O and N hard-donor sites, which can generate five- and six-membered rings upon coordination to metal ions, can be obtained from the condensation of a salicyl­aldehyde derivative and hydrazides (Pellerito & Nagy, 2002 ▸). Many research groups have designed hydrazone ligands to prepare metal complexes with particular properties. Thus, organotin(IV) complexes were synthesized from ligands having a hydrazone moiety. The anti­bacterial (Rehman *et al.*, 2016 ▸), anti­fungal (Öztaş *et al.*, 2009 ▸) and anti­tumour (Lee *et al.*, 2015 ▸) properties of these complexes have been studied. The structures of these organotin(IV) complexes and their properties can be diverse depending on the number of alkyl groups linked to Sn^4+^ (Lima *et al.*, 2015 ▸; Luna-García *et al.*, 2009 ▸). In this context, we have synthesized a symmetric ligand by a condensation reaction between salicyl­aldehyde and oxalohydrazide. This ligand was used to synthesize the organostannic(IV) complex, the structure of which is described herein.
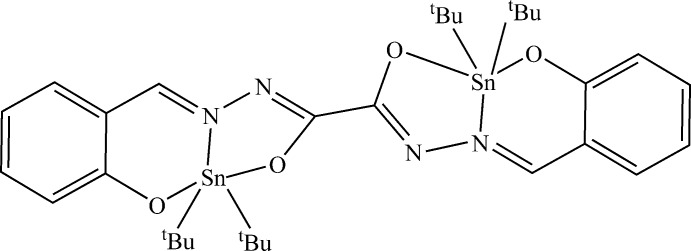


## Structural commentary   

**Figure 1:**
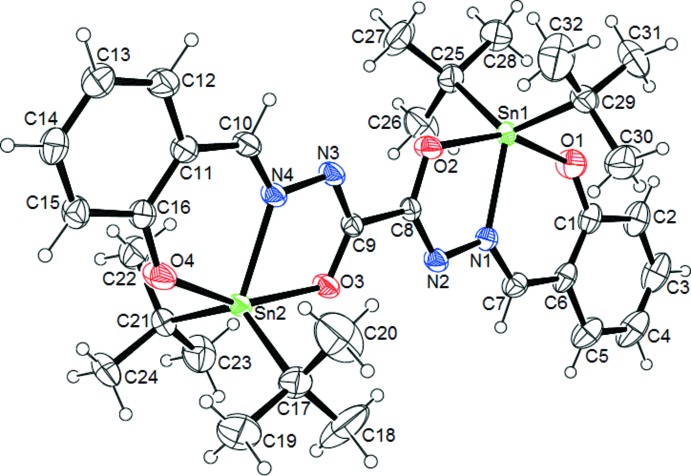
The mol­ecular structure of the title compound, showing the atom-numbering scheme. Displacement ellipsoids are plotted at the 50% probability level.

The structure of the title complex is shown in Fig. 1 ▸. The compound is a neutral pseudocentrosymmetric complex, which crystallizes in the *P*2_1_/*n* space group. In the asymmetric unit, one organic ligand links two [Sn(^*t*^Bu)_2_]^2+^ units in a tridentate fashion. The stannic units are connected by the doubly deprotonated ligand which play a bridging role in a *trans* conformation. Each stannic unit is coordinated to the ligand *via* an imino­late O atom, a phenolate O atom and an imine N atom. Each Sn atom is penta­coordinated. The Sn—C bond lengths [2.158 (3)–2.168 (3) Å] are slightly shorter than the values reported for complexes containing the [Sn(^*t*^Bu)_2_]^2+^ unit (Reichelt & Reuter, 2013 ▸, 2014 ▸). The binding lengths Sn—O_phenolate_ [2.0973 (18) and 2.0979 (18) Å, respectively, for Sn1 and Sn2] are shorter than the Sn—O_imino­late_ bond lengths [2.1497 (16) and 2.1633 (16) Å, respectively, for Sn1 and Sn2] (Table 1 ▸). The phenolate O atoms are more strongly coordinated to the Sn atom than the imino­late O atoms. Consequently, the respective C—O bond lengths are unequal: the C—O_phenolate_ distances associated with the strong coordination [1.302 (3)–1.308 (3) Å] are longer than the C—O_imino­late_ bonds associated with the less strong coordination [1.283 (3)–1.288 (3) Å]. The coordination sphere SnNC_2_O_2_ for each of the two Sn atoms can be characterized by the trigonality parameter τ = (β − α)/60, with α and β being the two largest angles around Sn (Addison *et al.*, 1984 ▸). The value of τ is 1 in the case of a trigonal bipyramidal geometry, whereas τ = 0 for a perfect square-based pyramid. In the case of our complex, the values of τ (0.44 for Sn1 and 0.41 for Sn2) indicate inter­mediate geometries between the two perfect environments. For the two Sn atoms, the comparison of the values of the angles found in the coordination sphere with the ideal values of the angles for trigonal bipyramidal geometry indicates that the environment around the Sn atoms is best described as a strongly distorted trigonal bipyramid. The bond angles between the *tert*-butyl groups around Sn [C—Sn—C = 128.35 (12)° for Sn1 and 130.02 (12)° for Sn2] result in compression of the bond angles with the third atom which forms the equatorial plane with the two *tert*-butyl groups [N—Sn—C = 113.85 (10) and 117.79 (10)° for Sn1, and 113.63 (11) and 116.29 (10)° for Sn2]. The sum of the angles in the basal planes are, respectively, 359.99° for Sn1 and 359.94° for Sn2. The O atoms occupy the apical positions with comparable angles of 154.61 (7)° for Sn1 and 154.73 (7)° for Sn2. The angles between the apical O atoms and the atoms in the basal plane are in the range 72.35 (7)–97.12 (11)° for Sn1 and between 72.39 (6) and 96.48 (9)° for Sn2. The ligand, which acts in a tridentate fashion, forms two rings upon coordination with the tin centres, *i.e.* a five-membered OCNNSn ring and a six-membered OCCCNSn ring, sharing atom N1 for Sn1 and N4 for Sn2. The angles resulting from the five-membered ring [N1—Sn1—O2 = 72.35 (7)° and N4—Sn2—O3 = 72.39 (6)°] are much smaller than the angles resulting from the six-membered ring [N1—Sn1—O1 = 82.32 (8)° and N4—Sn2—O4 = 82.39 (7)°]. The better flexibility of the six-membered ring can explain this observed difference in values. The five- and six-membered rings obtained after coordination of the ligand are not planar, as indicated by the torsion angles for the two Sn atoms in the complex: Sn1—N1—N2—C8 0.6, Sn1—O2—C8—N2 0.5, Sn1—O1—C1—C6 6.3, Sn1—N1—C7—C6 − 2, Sn2—N4—N3—C9 2.1, Sn2—O3—C9—N3 − 1.2, Sn2—O4—C16—C11 − 3.7 and Sn2—N4—C10—C11 − 0.5°. For all four ^*t*^Bu groups, the angles around the central C atom (Sn—C—C and C—C—C) vary in the range from 106.0 (3) to 112.3 (4)° and indicate a tetra­hedral environment around the central C atom. Both ^*t*^Bu groups reveal an eclipsed conformation regarding the methyl groups. The C—C bond lengths are in the range 1.81 (5)–1.542 (9) Å and are comparable to the values found in the literature (Reichelt & Reuter, 2013 ▸).

## Supra­molecular features   

**Figure 2:**
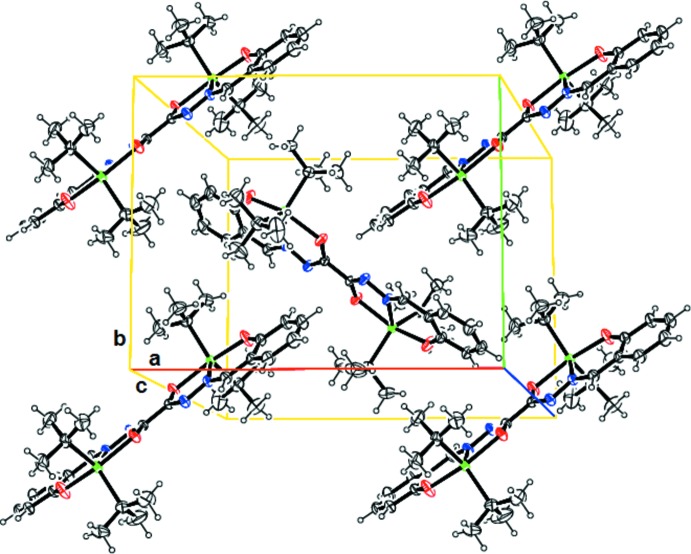
A view of the crystal packing of the title compound.

The overall structure is a complex three-dimensional network which is constructed from neutral quasi-centrosymmetric complexes disposed in different orientations onto inter­secting multilayers (Fig. 2 ▸). The complex mol­ecules display no strong supramolecular inter­actions and there are no hydrogen-bonding contacts in the crystal. This may be a consequence of a steric hindrance generated by the *tert*-butyl groups which could keep the complex mol­ecules distant from each other.

## Database survey   

No information was found in the databases for this ligand.

## Synthesis and crystallization   

To a solution of oxalyldihydrazine (1 mmol) in a mixture of water and methanol (1:3 *v*/*v*, 10 ml) was added a solution of salicyl­aldehyde (2 mmol) in 10 ml of the same mixture. A white precipitate appeared and the resulting mixture was stirred at room temperature for 24 h. The suspension was filtered and the solid was washed with 2 × 10 ml of water and 2 × 10 ml of ether. The solid was recrystallized from a mixture of chloro­form and methanol (1:1 *v*/*v*). The white powder collected was dried under P_2_O_5_. Yield 90% (H_4_ *L*). Calculated for C_16_H_14_N_4_O_4_: C 58.89, H 4.32, N 17.17%; found: C 59.02, H 4.37, N 17.24%. IR (cm^−1^): 3277 (ν O—H), 1664 (ν C=O), 1601 (ν C=N), 1533, 1486, 1457, 1357, 1304, 1259, 1218, 1161 (ν C—O), 776, 673. ^1^H NMR: δ 12.6 (2H, broad, H—O_phenolic_), 11.00 (*s*, 2H, broad, H—O_imino­lic_), 8.85 (*s*, 2H, broad, H—C=N), 7.6–7.00 (*mult*, 8H, H—Ph). ^13^C NMR: δ 158.5, 156.8, 151.98, 148.00, 132.93, 130.27, 120.37, 119.54, 117.39. To a mixture of H_4_ *L* (2 mmol) and tri­ethyl­amine (4 mmol) in 10 ml of ethanol was added SnCl_2_^*t*^Bu_2_ (2 mmol) in ethanol (10 ml). The resulting yellow mixture was stirred under reflux for 120 min and the resulting brown solution was filtered. The filtrate was kept at 298 K and after one week yellow crystals suitable for X-ray analysis appeared and were collected by filtration. Yield 40%, m.p. 243°C. Calculated for C_32_H_46_N_4_Sn_2_O_4_: C 48.77, H 5.88, N 7.11%; found: C 48.64, H 5.96, N 7.09%. IR (cm^−1^): 1609, 1537, 1516, 1468, 1441, 1367, 1310, 1275, 1198, 1167, 1150, 870, 771, 754. ^1^H NMR: δ 8.85 (*s*, 2H, broad, H—C=N); 7.13–6.69 (*mult*, 8H, H—Ph); 1.33 (*s*, 36H, –^*t*^Bu). ^13^C NMR: δ 168.80, 163.68, 135.85, 134.72, 122.22, 116.99, 41.53, 29.96.

## Refinement   

Crystal data, data collection and structure refinement details are summarized in Table 2 ▸. All H atoms were geometrically optimized and refined as riding, with *U*_iso_(H) = 1.2*U*_eq_(C) (1.5 for CH_3_ groups).

## Supplementary Material

Crystal structure: contains datablock(s) I. DOI: 10.1107/S2056989018007077/ex2008sup1.cif

Structure factors: contains datablock(s) I. DOI: 10.1107/S2056989018007077/ex2008Isup2.hkl

CCDC reference: 1842349

Additional supporting information:  crystallographic information; 3D view; checkCIF report

## Figures and Tables

**Table 1:** Selected geometric parameters (Å, °)

Sn1—O1	2.0973 (18)	Sn2—O4	2.0979 (18)
Sn1—O2	2.1497 (16)	Sn2—O3	2.1633 (16)
Sn1—C29	2.158 (3)	Sn2—C17	2.166 (3)
Sn1—C25	2.163 (3)	Sn2—C21	2.168 (3)
Sn1—N1	2.1855 (19)	Sn2—N4	2.1840 (19)
			
O1—Sn1—O2	154.61 (7)	O4—Sn2—O3	154.73 (7)
C29—Sn1—C25	128.35 (12)	C17—Sn2—C21	130.02 (12)
C29—Sn1—N1	113.85 (10)	C17—Sn2—N4	113.63 (11)
C25—Sn1—N1	117.79 (10)	C21—Sn2—N4	116.29 (10)

**Table 2:** Experimental details

Crystal data	
Chemical formula	[Sn_2_(C_4_H_9_)_4_(C_16_H_10_N_4_O_4_)]
*M*_r_	788.11
Crystal system, space group	Monoclinic, *P*2_1_/*n*
Temperature (K)	293
*a*, *b*, *c* (Å)	16.3836 (8), 13.2683 (9), 16.8153 (9)
β (°)	101.829 (5)
*V* (Å^3^)	3577.7 (4)
*Z*	4
Radiation type	Mo *K*α
μ (mm^−1^)	1.43
Crystal size (mm)	0.12 × 0.09 × 0.07

Data collection	
Diffractometer	Nonius KappaCCD
No. of measured, independent and observed [*I* > 2σ(*I*)] reflections	59628, 9468, 7650
*R*_int_	0.048
(sin θ/λ)_max_ (Å^−1^)	0.702

Refinement	
*R*[*F* ^2^ > 2σ(*F* ^2^)], *wR*(*F* ^2^), *S*	0.030, 0.073, 1.04
No. of reflections	9468
No. of parameters	379
H-atom treatment	H atoms treated by a mixture of independent and constrained refinement
Δρ_max_, Δρ_min_ (e Å^−3^)	0.45, −0.71

Computer programs: *APEX3* and *SAINT* (Bruker, 2016 ▸), *SHELXT* (Sheldrick, 2015*a* ▸), *SHELXL2014* (Sheldrick, 2015*b* ▸) and *ORTEP-3 for Windows* (Farrugia, 2012 ▸).

## supplementary crystallographic information

### Crystal data

**Table:**

[Sn_2_(C_4_H_9_)_4_(C_16_H_10_N_4_O_4_)]	*F*(000) = 1592
*M**_r_* = 788.11	*D*_x_ = 1.463 Mg m^−^^3^*D*_m_ = not messured Mg m^−^^3^*D*_m_ measured by ?
Monoclinic, *P*2_1_/*n*	Mo *K*α radiation, λ = 0.71073 Å
*a* = 16.3836 (8) Å	Cell parameters from 4920 reflections
*b* = 13.2683 (9) Å	θ = 2.4–28.6°
*c* = 16.8153 (9) Å	µ = 1.43 mm^−^^1^
β = 101.829 (5)°	*T* = 293 K
*V* = 3577.7 (4) Å^3^	Block, colourless
*Z* = 4	0.12 × 0.09 × 0.07 mm

### Data collection

**Table:**

Nonius KappaCCD diffractometer	7650 reflections with *I* > 2σ(*I*)
Radiation source: fine-focus sealed tube	*R*_int_ = 0.048
Detector resolution: 9 pixels mm^-1^	θ_max_ = 29.9°, θ_min_ = 3.4°
CCD scans	*h* = −22→22
59628 measured reflections	*k* = −17→18
9468 independent reflections	*l* = −20→21

### Refinement

**Table:**

Refinement on *F*^2^	Primary atom site location: structure-invariant direct methods
Least-squares matrix: full	Secondary atom site location: difference Fourier map
*R*[*F*^2^ > 2σ(*F*^2^)] = 0.030	Hydrogen site location: inferred from neighbouring sites
*wR*(*F*^2^) = 0.073	H atoms treated by a mixture of independent and constrained refinement
*S* = 1.04	*w* = 1/[σ^2^(*F*_o_^2^) + (0.0319*P*)^2^ + 1.6129*P*] where *P* = (*F*_o_^2^ + 2*F*_c_^2^)/3
9468 reflections	(Δ/σ)_max_ = 0.002
379 parameters	Δρ_max_ = 0.45 e Å^−^^3^
0 restraints	Δρ_min_ = −0.71 e Å^−^^3^

### Special details

**Table:**

Geometry. All esds (except the esd in the dihedral angle between two l.s. planes) are estimated using the full covariance matrix. The cell esds are taken into account individually in the estimation of esds in distances, angles and torsion angles; correlations between esds in cell parameters are only used when they are defined by crystal symmetry. An approximate (isotropic) treatment of cell esds is used for estimating esds involving l.s. planes.

### Fractional atomic coordinates and isotropic or equivalent isotropic displacement parameters (Å^2^)

**Table:**

	*x*	*y*	*z*	*U*_iso_*/*U*_eq_	
Sn1	0.36223 (2)	0.41469 (2)	0.79310 (2)	0.03755 (5)	
Sn2	0.63988 (2)	0.79558 (2)	0.68119 (2)	0.03934 (5)	
O1	0.24901 (12)	0.34843 (18)	0.73636 (14)	0.0703 (6)	
O2	0.46637 (11)	0.51524 (15)	0.80024 (11)	0.0525 (5)	
O3	0.53626 (11)	0.69220 (14)	0.67204 (10)	0.0474 (4)	
O4	0.75082 (13)	0.86525 (18)	0.74008 (12)	0.0694 (7)	
N1	0.34011 (12)	0.50542 (15)	0.68228 (11)	0.0393 (4)	
N2	0.40219 (13)	0.57578 (16)	0.67497 (12)	0.0428 (4)	
N3	0.58678 (13)	0.65449 (16)	0.80715 (12)	0.0442 (5)	
N4	0.65020 (12)	0.72276 (16)	0.79931 (12)	0.0414 (4)	
C1	0.19341 (15)	0.3673 (2)	0.67079 (18)	0.0528 (6)	
C2	0.11932 (19)	0.3098 (3)	0.6553 (2)	0.0711 (9)	
H2	0.110885	0.261020	0.692523	0.085*	
C3	0.0599 (2)	0.3247 (3)	0.5865 (3)	0.0853 (12)	
H3	0.011867	0.285444	0.577350	0.102*	
C4	0.0698 (2)	0.3965 (3)	0.5307 (3)	0.0850 (12)	
H4	0.028528	0.405890	0.484257	0.102*	
C5	0.14110 (19)	0.4550 (3)	0.5433 (2)	0.0686 (9)	
H5	0.147829	0.503344	0.505177	0.082*	
C6	0.20378 (15)	0.4417 (2)	0.61383 (17)	0.0503 (6)	
C7	0.27601 (16)	0.5052 (2)	0.62256 (15)	0.0471 (6)	
H7	0.276901	0.551011	0.580904	0.056*	
C8	0.46227 (13)	0.57313 (16)	0.73846 (13)	0.0344 (4)	
C9	0.53356 (13)	0.64555 (17)	0.73865 (13)	0.0356 (4)	
C10	0.70722 (16)	0.7344 (2)	0.86415 (15)	0.0493 (6)	
H10	0.700861	0.696916	0.909235	0.059*	
C11	0.77885 (16)	0.7980 (2)	0.87465 (15)	0.0465 (6)	
C12	0.83292 (19)	0.7991 (3)	0.95231 (17)	0.0619 (8)	
H12	0.819572	0.760608	0.994057	0.074*	
C13	0.90410 (18)	0.8556 (3)	0.96701 (18)	0.0655 (8)	
H13	0.938677	0.855760	1.018321	0.079*	
C14	0.92427 (17)	0.9124 (2)	0.90516 (19)	0.0567 (7)	
H14	0.973175	0.950086	0.914915	0.068*	
C15	0.87323 (16)	0.9143 (2)	0.82924 (18)	0.0506 (6)	
H15	0.888326	0.952862	0.788380	0.061*	
C16	0.79855 (15)	0.8587 (2)	0.81248 (15)	0.0450 (5)	
C17	0.5658 (2)	0.9320 (2)	0.6679 (2)	0.0669 (8)	
C18	0.4847 (3)	0.9143 (4)	0.6085 (4)	0.142 (3)	
H18A	0.454931	0.860176	0.627670	0.213*	
H18B	0.451533	0.974447	0.603517	0.213*	
H18C	0.496052	0.896819	0.556398	0.213*	
C19	0.6150 (3)	1.0174 (3)	0.6397 (3)	0.0981 (14)	
H19A	0.666229	1.027155	0.678515	0.147*	
H19B	0.627256	1.000674	0.587824	0.147*	
H19C	0.582737	1.078302	0.634943	0.147*	
C20	0.5514 (4)	0.9567 (4)	0.7526 (4)	0.137 (2)	
H20A	0.604119	0.967599	0.788836	0.206*	
H20B	0.518037	1.016579	0.750218	0.206*	
H20C	0.523032	0.901603	0.772148	0.206*	
C21	0.69982 (17)	0.7192 (2)	0.59457 (17)	0.0540 (7)	
C22	0.7587 (2)	0.6409 (3)	0.6418 (2)	0.0801 (10)	
H22A	0.727044	0.592205	0.665022	0.120*	
H22B	0.788490	0.607630	0.605812	0.120*	
H22C	0.797641	0.673657	0.684458	0.120*	
C23	0.6346 (2)	0.6689 (3)	0.5285 (2)	0.0817 (11)	
H23A	0.603860	0.620308	0.552904	0.122*	
H23B	0.597028	0.719105	0.500825	0.122*	
H23C	0.661852	0.635758	0.490429	0.122*	
C24	0.7494 (2)	0.7965 (3)	0.5577 (2)	0.0803 (11)	
H24A	0.712025	0.845560	0.528066	0.121*	
H24B	0.788287	0.829454	0.600229	0.121*	
H24C	0.779136	0.763427	0.521582	0.121*	
C25	0.43127 (19)	0.2757 (2)	0.7933 (2)	0.0578 (7)	
C26	0.4308 (3)	0.2502 (3)	0.7050 (3)	0.0927 (13)	
H26A	0.374299	0.242582	0.675844	0.139*	
H26B	0.457188	0.303542	0.681016	0.139*	
H26C	0.460630	0.188421	0.702427	0.139*	
C27	0.5201 (2)	0.2885 (3)	0.8417 (3)	0.0956 (14)	
H27A	0.518815	0.304510	0.897070	0.143*	
H27B	0.550483	0.226908	0.839912	0.143*	
H27C	0.547041	0.342028	0.818502	0.143*	
C28	0.3865 (2)	0.1936 (2)	0.8315 (2)	0.0755 (10)	
H28A	0.387192	0.210408	0.887113	0.113*	
H28B	0.329886	0.188329	0.802171	0.113*	
H28C	0.414414	0.130347	0.829091	0.113*	
C29	0.3129 (2)	0.4803 (2)	0.89081 (17)	0.0596 (7)	
C30	0.2509 (3)	0.5633 (3)	0.8534 (3)	0.0962 (14)	
H30A	0.208371	0.534604	0.811715	0.144*	
H30B	0.225771	0.592226	0.894872	0.144*	
H30C	0.280030	0.614798	0.830230	0.144*	
C31	0.2614 (3)	0.4006 (4)	0.9233 (3)	0.1040 (15)	
H31A	0.218264	0.376591	0.879876	0.156*	
H31B	0.296795	0.345402	0.945335	0.156*	
H31C	0.236661	0.429417	0.965076	0.156*	
C32	0.3804 (3)	0.5197 (5)	0.9557 (3)	0.122 (2)	
H32A	0.411643	0.569682	0.933459	0.183*	
H32B	0.356563	0.549440	0.997807	0.183*	
H32C	0.416697	0.465424	0.978065	0.183*	

### Atomic displacement parameters (Å^2^)

**Table:**

	*U*^11^	*U*^22^	*U*^33^	*U*^12^	*U*^13^	*U*^23^
Sn1	0.03209 (8)	0.03814 (9)	0.04242 (9)	−0.00636 (6)	0.00766 (6)	0.00305 (6)
Sn2	0.03622 (9)	0.04356 (10)	0.03705 (9)	−0.00783 (6)	0.00475 (6)	0.01043 (6)
O1	0.0483 (11)	0.0805 (16)	0.0749 (14)	−0.0318 (11)	−0.0044 (10)	0.0142 (12)
O2	0.0436 (9)	0.0602 (12)	0.0478 (9)	−0.0227 (8)	−0.0045 (7)	0.0194 (8)
O3	0.0447 (9)	0.0569 (11)	0.0384 (8)	−0.0174 (8)	0.0033 (7)	0.0114 (8)
O4	0.0593 (12)	0.0872 (16)	0.0530 (11)	−0.0384 (11)	−0.0086 (9)	0.0260 (11)
N1	0.0336 (9)	0.0424 (11)	0.0407 (10)	−0.0046 (8)	0.0052 (8)	0.0004 (8)
N2	0.0397 (10)	0.0473 (12)	0.0406 (10)	−0.0100 (9)	0.0064 (8)	0.0061 (8)
N3	0.0415 (10)	0.0494 (12)	0.0406 (10)	−0.0155 (9)	0.0057 (8)	0.0106 (9)
N4	0.0407 (10)	0.0445 (11)	0.0381 (10)	−0.0118 (8)	0.0056 (8)	0.0077 (8)
C1	0.0349 (12)	0.0554 (16)	0.0661 (17)	−0.0053 (11)	0.0055 (11)	−0.0130 (13)
C2	0.0411 (15)	0.074 (2)	0.095 (2)	−0.0147 (14)	0.0064 (15)	−0.0158 (18)
C3	0.0419 (16)	0.085 (3)	0.120 (3)	−0.0102 (17)	−0.0045 (18)	−0.035 (2)
C4	0.0466 (17)	0.096 (3)	0.095 (3)	0.0031 (18)	−0.0275 (18)	−0.033 (2)
C5	0.0541 (17)	0.072 (2)	0.0687 (19)	0.0045 (15)	−0.0143 (14)	−0.0171 (16)
C6	0.0339 (12)	0.0562 (16)	0.0563 (15)	0.0024 (11)	−0.0018 (11)	−0.0197 (12)
C7	0.0432 (13)	0.0523 (15)	0.0422 (12)	−0.0004 (11)	0.0008 (10)	−0.0024 (11)
C8	0.0319 (10)	0.0343 (11)	0.0384 (11)	−0.0024 (8)	0.0105 (8)	0.0001 (9)
C9	0.0342 (10)	0.0352 (11)	0.0387 (11)	−0.0028 (9)	0.0107 (9)	0.0028 (9)
C10	0.0475 (14)	0.0603 (16)	0.0377 (12)	−0.0163 (12)	0.0032 (10)	0.0105 (11)
C11	0.0419 (13)	0.0537 (15)	0.0414 (13)	−0.0096 (11)	0.0029 (10)	0.0020 (11)
C12	0.0564 (17)	0.082 (2)	0.0424 (14)	−0.0167 (15)	−0.0019 (12)	0.0067 (13)
C13	0.0504 (16)	0.088 (2)	0.0518 (16)	−0.0162 (16)	−0.0053 (12)	−0.0062 (15)
C14	0.0402 (13)	0.0623 (18)	0.0650 (17)	−0.0119 (12)	0.0046 (12)	−0.0111 (14)
C15	0.0422 (13)	0.0506 (15)	0.0585 (16)	−0.0122 (11)	0.0094 (11)	−0.0020 (12)
C16	0.0397 (12)	0.0462 (14)	0.0469 (13)	−0.0093 (10)	0.0041 (10)	0.0006 (10)
C17	0.0647 (19)	0.0472 (16)	0.089 (2)	0.0057 (14)	0.0171 (17)	0.0156 (15)
C18	0.072 (3)	0.083 (3)	0.240 (7)	0.017 (2)	−0.041 (4)	0.041 (4)
C19	0.111 (3)	0.050 (2)	0.133 (4)	−0.002 (2)	0.027 (3)	0.031 (2)
C20	0.201 (6)	0.079 (3)	0.164 (5)	0.042 (4)	0.114 (5)	0.011 (3)
C21	0.0475 (14)	0.0667 (18)	0.0516 (15)	−0.0002 (13)	0.0194 (12)	0.0093 (13)
C22	0.068 (2)	0.078 (2)	0.100 (3)	0.0197 (18)	0.032 (2)	0.022 (2)
C23	0.084 (2)	0.103 (3)	0.0611 (19)	−0.005 (2)	0.0222 (18)	−0.0192 (19)
C24	0.077 (2)	0.097 (3)	0.080 (2)	−0.0037 (19)	0.0441 (19)	0.025 (2)
C25	0.0543 (16)	0.0437 (15)	0.078 (2)	0.0058 (12)	0.0202 (14)	0.0083 (13)
C26	0.127 (4)	0.066 (2)	0.100 (3)	0.010 (2)	0.059 (3)	−0.008 (2)
C27	0.0511 (19)	0.082 (3)	0.149 (4)	0.0168 (18)	0.008 (2)	0.035 (3)
C28	0.082 (2)	0.0448 (17)	0.102 (3)	−0.0003 (16)	0.025 (2)	0.0177 (17)
C29	0.0664 (18)	0.0682 (19)	0.0484 (15)	0.0080 (15)	0.0212 (13)	0.0051 (13)
C30	0.114 (3)	0.099 (3)	0.078 (2)	0.046 (3)	0.025 (2)	0.002 (2)
C31	0.105 (3)	0.128 (4)	0.096 (3)	0.001 (3)	0.062 (3)	0.023 (3)
C32	0.085 (3)	0.200 (6)	0.078 (3)	0.001 (3)	0.009 (2)	−0.058 (3)

### Geometric parameters (Å, º)

**Table:**

Sn1—O1	2.0973 (18)	C17—C20	1.527 (6)
Sn1—O2	2.1497 (16)	C18—H18A	0.9600
Sn1—C29	2.158 (3)	C18—H18B	0.9600
Sn1—C25	2.163 (3)	C18—H18C	0.9600
Sn1—N1	2.1855 (19)	C19—H19A	0.9600
Sn2—O4	2.0979 (18)	C19—H19B	0.9600
Sn2—O3	2.1633 (16)	C19—H19C	0.9600
Sn2—C17	2.166 (3)	C20—H20A	0.9600
Sn2—C21	2.168 (3)	C20—H20B	0.9600
Sn2—N4	2.1840 (19)	C20—H20C	0.9600
O1—C1	1.302 (3)	C21—C24	1.517 (4)
O2—C8	1.283 (3)	C21—C22	1.525 (4)
O3—C9	1.288 (3)	C21—C23	1.528 (4)
O4—C16	1.308 (3)	C22—H22A	0.9600
N1—C7	1.296 (3)	C22—H22B	0.9600
N1—N2	1.404 (3)	C22—H22C	0.9600
N2—C8	1.296 (3)	C23—H23A	0.9600
N3—C9	1.300 (3)	C23—H23B	0.9600
N3—N4	1.405 (3)	C23—H23C	0.9600
N4—C10	1.291 (3)	C24—H24A	0.9600
C1—C6	1.410 (4)	C24—H24B	0.9600
C1—C2	1.412 (4)	C24—H24C	0.9600
C2—C3	1.365 (5)	C25—C26	1.522 (5)
C2—H2	0.9300	C25—C27	1.524 (5)
C3—C4	1.370 (6)	C25—C28	1.526 (4)
C3—H3	0.9300	C26—H26A	0.9600
C4—C5	1.382 (5)	C26—H26B	0.9600
C4—H4	0.9300	C26—H26C	0.9600
C5—C6	1.411 (4)	C27—H27A	0.9600
C5—H5	0.9300	C27—H27B	0.9600
C6—C7	1.435 (4)	C27—H27C	0.9600
C7—H7	0.9300	C28—H28A	0.9600
C8—C9	1.512 (3)	C28—H28B	0.9600
C10—C11	1.427 (3)	C28—H28C	0.9600
C10—H10	0.9300	C29—C32	1.481 (5)
C11—C16	1.409 (4)	C29—C31	1.523 (5)
C11—C12	1.420 (3)	C29—C30	1.542 (5)
C12—C13	1.365 (4)	C30—H30A	0.9600
C12—H12	0.9300	C30—H30B	0.9600
C13—C14	1.378 (4)	C30—H30C	0.9600
C13—H13	0.9300	C31—H31A	0.9600
C14—C15	1.375 (4)	C31—H31B	0.9600
C14—H14	0.9300	C31—H31C	0.9600
C15—C16	1.406 (3)	C32—H32A	0.9600
C15—H15	0.9300	C32—H32B	0.9600
C17—C18	1.508 (6)	C32—H32C	0.9600
C17—C19	1.522 (5)		
			
O1—Sn1—O2	154.61 (7)	H18A—C18—H18B	109.5
O1—Sn1—C29	94.65 (11)	C17—C18—H18C	109.5
O2—Sn1—C29	97.12 (11)	H18A—C18—H18C	109.5
O1—Sn1—C25	93.27 (11)	H18B—C18—H18C	109.5
O2—Sn1—C25	96.90 (10)	C17—C19—H19A	109.5
C29—Sn1—C25	128.35 (12)	C17—C19—H19B	109.5
O1—Sn1—N1	82.32 (8)	H19A—C19—H19B	109.5
O2—Sn1—N1	72.35 (7)	C17—C19—H19C	109.5
C29—Sn1—N1	113.85 (10)	H19A—C19—H19C	109.5
C25—Sn1—N1	117.79 (10)	H19B—C19—H19C	109.5
O4—Sn2—O3	154.73 (7)	C17—C20—H20A	109.5
O4—Sn2—C17	95.37 (12)	C17—C20—H20B	109.5
O3—Sn2—C17	96.17 (10)	H20A—C20—H20B	109.5
O4—Sn2—C21	93.16 (11)	C17—C20—H20C	109.5
O3—Sn2—C21	96.48 (9)	H20A—C20—H20C	109.5
C17—Sn2—C21	130.02 (12)	H20B—C20—H20C	109.5
O4—Sn2—N4	82.39 (7)	C24—C21—C22	109.8 (3)
O3—Sn2—N4	72.39 (6)	C24—C21—C23	110.6 (3)
C17—Sn2—N4	113.63 (11)	C22—C21—C23	110.8 (3)
C21—Sn2—N4	116.29 (10)	C24—C21—Sn2	108.2 (2)
C1—O1—Sn1	134.77 (19)	C22—C21—Sn2	107.1 (2)
C8—O2—Sn1	114.66 (14)	C23—C21—Sn2	110.3 (2)
C9—O3—Sn2	114.15 (14)	C21—C22—H22A	109.5
C16—O4—Sn2	134.83 (17)	C21—C22—H22B	109.5
C7—N1—N2	114.9 (2)	H22A—C22—H22B	109.5
C7—N1—Sn1	128.74 (17)	C21—C22—H22C	109.5
N2—N1—Sn1	116.38 (13)	H22A—C22—H22C	109.5
C8—N2—N1	110.57 (18)	H22B—C22—H22C	109.5
C9—N3—N4	110.51 (18)	C21—C23—H23A	109.5
C10—N4—N3	114.75 (19)	C21—C23—H23B	109.5
C10—N4—Sn2	128.52 (16)	H23A—C23—H23B	109.5
N3—N4—Sn2	116.73 (14)	C21—C23—H23C	109.5
O1—C1—C6	123.3 (2)	H23A—C23—H23C	109.5
O1—C1—C2	118.5 (3)	H23B—C23—H23C	109.5
C6—C1—C2	118.2 (3)	C21—C24—H24A	109.5
C3—C2—C1	121.0 (4)	C21—C24—H24B	109.5
C3—C2—H2	119.5	H24A—C24—H24B	109.5
C1—C2—H2	119.5	C21—C24—H24C	109.5
C2—C3—C4	121.2 (3)	H24A—C24—H24C	109.5
C2—C3—H3	119.4	H24B—C24—H24C	109.5
C4—C3—H3	119.4	C26—C25—C27	111.0 (3)
C3—C4—C5	120.0 (3)	C26—C25—C28	110.1 (3)
C3—C4—H4	120.0	C27—C25—C28	110.1 (3)
C5—C4—H4	120.0	C26—C25—Sn1	106.9 (2)
C4—C5—C6	120.4 (4)	C27—C25—Sn1	110.4 (2)
C4—C5—H5	119.8	C28—C25—Sn1	108.3 (2)
C6—C5—H5	119.8	C25—C26—H26A	109.5
C1—C6—C5	119.3 (3)	C25—C26—H26B	109.5
C1—C6—C7	123.6 (2)	H26A—C26—H26B	109.5
C5—C6—C7	117.1 (3)	C25—C26—H26C	109.5
N1—C7—C6	126.9 (3)	H26A—C26—H26C	109.5
N1—C7—H7	116.5	H26B—C26—H26C	109.5
C6—C7—H7	116.5	C25—C27—H27A	109.5
O2—C8—N2	126.0 (2)	C25—C27—H27B	109.5
O2—C8—C9	117.82 (19)	H27A—C27—H27B	109.5
N2—C8—C9	116.1 (2)	C25—C27—H27C	109.5
O3—C9—N3	126.2 (2)	H27A—C27—H27C	109.5
O3—C9—C8	117.89 (19)	H27B—C27—H27C	109.5
N3—C9—C8	115.94 (19)	C25—C28—H28A	109.5
N4—C10—C11	127.5 (2)	C25—C28—H28B	109.5
N4—C10—H10	116.2	H28A—C28—H28B	109.5
C11—C10—H10	116.2	C25—C28—H28C	109.5
C16—C11—C12	118.8 (2)	H28A—C28—H28C	109.5
C16—C11—C10	123.7 (2)	H28B—C28—H28C	109.5
C12—C11—C10	117.5 (2)	C32—C29—C31	111.4 (4)
C13—C12—C11	121.4 (3)	C32—C29—C30	112.3 (4)
C13—C12—H12	119.3	C31—C29—C30	106.0 (3)
C11—C12—H12	119.3	C32—C29—Sn1	111.3 (2)
C12—C13—C14	119.4 (3)	C31—C29—Sn1	108.3 (2)
C12—C13—H13	120.3	C30—C29—Sn1	107.2 (2)
C14—C13—H13	120.3	C29—C30—H30A	109.5
C15—C14—C13	121.1 (3)	C29—C30—H30B	109.5
C15—C14—H14	119.5	H30A—C30—H30B	109.5
C13—C14—H14	119.5	C29—C30—H30C	109.5
C14—C15—C16	121.0 (3)	H30A—C30—H30C	109.5
C14—C15—H15	119.5	H30B—C30—H30C	109.5
C16—C15—H15	119.5	C29—C31—H31A	109.5
O4—C16—C15	118.8 (2)	C29—C31—H31B	109.5
O4—C16—C11	123.0 (2)	H31A—C31—H31B	109.5
C15—C16—C11	118.2 (2)	C29—C31—H31C	109.5
C18—C17—C19	111.2 (4)	H31A—C31—H31C	109.5
C18—C17—C20	111.7 (4)	H31B—C31—H31C	109.5
C19—C17—C20	108.9 (4)	C29—C32—H32A	109.5
C18—C17—Sn2	109.7 (3)	C29—C32—H32B	109.5
C19—C17—Sn2	109.4 (2)	H32A—C32—H32B	109.5
C20—C17—Sn2	105.9 (3)	C29—C32—H32C	109.5
C17—C18—H18A	109.5	H32A—C32—H32C	109.5
C17—C18—H18B	109.5	H32B—C32—H32C	109.5
